# The mitochondrial genome of *Trichoclinocera yixianensis* (Diptera: Empididae)

**DOI:** 10.1080/23802359.2018.1462125

**Published:** 2018-04-12

**Authors:** Shang Gao, Jiale Zhou, Yike Cao, Junhua Zhang, Ding Yang

**Affiliations:** a College of Plant Protection, China Agricultural University, Beijing, China;; b Institute of Plant Quarantine, Chinese Academy of Inspection and Quarantine, Beijing, China

**Keywords:** Mitochondrial genome, Clinocerinae, phylogenetics

## Abstract

The dance fly *Trichoclinocera yixianensis* Li et Yang belongs to the subfamily Clinocerinae of Empididae. The mitogenome of *T. yixianensis* was sequenced, the first representative of the mitogenome of the subfamily. The nearly complete mitogenome is 15,595 bp totally, consisting of 13 protein-coding genes, 2 rRNAs, and 22 transfer RNAs. All genes have the similar locations and strands with that of other published species of Empididae. The nucleotide composition biases toward A and T, which together made up 80.1% of the entirety. Bayesian inference analysis strongly supported the monophyly of Empididae and Dolichopodidae. It suggested that Empididae is the sister group to the clade of Dolichopodidae and Empidinae is the sister group to the clade of Clinocerinae + Trichopezinae.

## Introduction

Empididae is one of the largest families in Diptera with over 5000 described species from the world (Yang et al. 2007). They capture arthropod prey, such as aphids, psyllids and coccids of Hemiptera, but also other true flies such as mosquitos, blackflies and so on. They are widely used as a biological indicator of evaluating the quality of environment and biodiversity (Yang and Yang 2003).

The specimens of *T. yixianensis* used for this study were deposited in the Entomological Museum of China Agricultural University (CAU). The total genomic DNA was extracted from the whole body (except head) of the specimen using the QIAamp DNA Blood Mini Kit (Qiagen, Germany) and stored at −20 °C until needed. The mitogenome was amplified and sequenced as described in our previous study (Wang, Li, Ding, et al. 2016). The nearly complete mitogenome of *T. yixianensis* is 15,595 bp. It encoded 13 PCGs, 22 tRNA genes, and 2 rRNA genes and the control region could not be sequenced entirely in this study, and were similar with related reports before (Kang et al. 2016; Li et al. 2016; Wang, Ding, et al. 2016; Wang, Wang, et al. 2016; Li et al. 2017; Zhou et al. 2017; Gao et al. 2018). All genes have the similar locations and strands with that of other published Empididae species. The nucleotide composition of the mitogenome was biased towards A and T, with 80.1% of A + T content (A = 41.6%, T = 38.5%, C =11.7%, G = 8.2%). The A + T content of PCGs, tRNAs, and rRNAs is 78.5%, 80.5%, and 82.3% respectively. The total length of all 13 PCGs of *T. yixianensis* is 11,240 bp. Four PCGs (*ATP8*, *NAD2*, *NAD5*, and *NAD6*) initiated with ATT codons, and five PCGs (*COII*, *COIII*, *ATP6*, *NAD4*, and *CYTB*) initiated with ATG codons, *NAD3* and *NAD4L* initiated with ATA as a start codon, *CO1* and *NAD1* initiated with TCG and TTG as a start codon, respectively. Ten PCGs used the typical termination codons TAA, two PCGs (*CO1* and *NAD4*) used T, and one PCG (*ND3*) used TAG in *T. yixianensis*.

**Figure 1.**
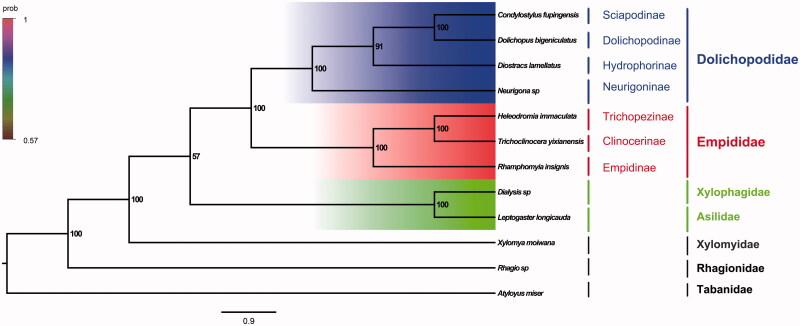
Bayesian phylogenetic tree of 12 Diptera species. The posterior probabilities are labeled at each node.

Phylogenetic analysis was performed based on the nucleotide sequences of 13 PCGs from 12 Diptera species. Bayesian (BI) analysis generated the phylogenetic tree topologies based on the PCGs matrices (Figure 1). According to the phylogenetic result, it showed that monophyletic Empidoidea was assigned to be the sister group to the clade of Xylophagidae and Asilidae. The monophyletic Empididae was assigned to the sister to the monophyletic Dolichopodidae. For the phylogeny of Empididae, the Empidinae is the sister group to the clade of Clinocerinae + Trichopezinae. The phylogenetic relationship within Empidoidea is very clear: Dolichopodidae + (Empidinae + (Clinocerinae + Trichopezinae)). The relationship of sister group between Empididae and Dolichopodidae was also supported by the previous study (Wang, Li, Ding, et al. 2016). The mitogenome of *T. yixianensis* could provide the important information for further studies of Empidoidea phylogeny.
